# Loss of Iroquois homeobox transcription factors 3 and 5 in osteoblasts disrupts cranial mineralization

**DOI:** 10.1016/j.bonr.2016.02.005

**Published:** 2016-04-13

**Authors:** Corey J. Cain, Nathalie Gaborit, Wint Lwin, Emilie Barruet, Samantha Ho, Carine Bonnard, Hanan Hamamy, Mohammad Shboul, Bruno Reversade, Hülya Kayserili, Benoit G. Bruneau, Edward C. Hsiao

**Affiliations:** a Department of Medicine, Division of Endocrinology and Metabolism, Institute for Human Genetics, Program in Craniofacial Biology, University of California, San Francisco, San Francisco, CA 94143-0794, USA; b Inserm, UMR 1087, l’institut du thorax, Nantes, France; c CNRS, UMR 6291, Nantes, France; d Université de Nantes, France; e Human Embryology and Genetics Laboratory, Institute of Medical Biology, A*STAR, Singapore 138648, Singapore; f Department of Genetic Medicine and Development, Geneva University, Geneva 1211, Switzerland; g Medical Genetics Department, Koc University School of Medicine, Rumelifeneri Yolu, Sarıyer, Istanbul 34450, Turkey; h Medical Genetics Department, Istanbul Medical Faculty, Istanbul University Topkapi, Fatih, 34093 lstanbul, Turkey; i Gladstone Institute for Cardiovascular Disease, San Francisco, CA 94158, USA; j Department of Pediatrics, University of California, San Francisco, San Francisco, CA 94143, USA

**Keywords:** Osteoclast/osteoblast biology, Osteoporosis, Osteoblast mineralization

## Abstract

Cranial malformations are a significant cause of perinatal morbidity and mortality. Iroquois homeobox transcription factors (IRX) are expressed early in bone tissue formation and facilitate patterning and mineralization of the skeleton. Mice lacking *Irx*5 appear grossly normal, suggesting that redundancy within the Iroquois family. However, global loss of both *Irx*3 and *Irx*5 in mice leads to significant skeletal malformations and embryonic lethality from cardiac defects. Here, we study the bone-specific functions of *Irx*3 and *Irx*5 using *Osx-Cre* to drive osteoblast lineage–specific deletion of *Irx*3 in *Irx*5^−/−^ mice. Although we found that the *Osx-Cre* transgene alone could also affect craniofacial mineralization, newborn *Irx*3^*flox*/*flox*^/*Irx*5^−/−^/*Osx-Cre*^+^ mice displayed additional mineralization defects in parietal, interparietal, and frontal bones with enlarged sutures and reduced calvarial expression of osteogenic genes. Newborn endochondral long bones were largely unaffected, but we observed marked reductions in 3–4-week old bone mineral content of *Irx*3^*flox*/*flox*^/*Irx*5^−/−^/*Osx-Cre*^+^ mice. Our findings indicate that IRX3 and IRX5 can work together to regulate mineralization of specific cranial bones. Our results also provide insight into the causes of the skeletal changes and mineralization defects seen in Hamamy syndrome patients carrying mutations in IRX5.

## Highlights

• Osteoblas*t*-specific deletion of *Irx*3 and *Irx*5 causes mineralization defects in parietal, interparietal, and frontal bones of newborn mice.
• 3–4 week old *Irx*3^*flox*/*flox*^/*Irx*5^−/−^/*Osx-Cre*^+^ mice show similar bone fragility to Hamamy syndrome patients who have mutations in IRX5.
• *Osx-Cre* transgene alone causes an early bone mineralization phenotype that recovers later.

## 1 Introduction

Craniofacial development requires tight coordination of cell migration, proliferation, and mineralization of osteogenic lineages (Wilkie and Morriss-Kay, 2001, Franz-Odendaal, 2011). Osteoblast dysfunction is thought to be a major contributor to diseases that affect craniofacial bones and mineralization (Rice, 2008). The complex genetic and spatial interactions that occur during craniofacial development pose major challenges to understanding mineralization during the development of the skull.

Iroquois homeobox domain transcription factors (IRX) are highly conserved proteins that regulate neural, cardiac, and bone development (Kerner et al., 2009, Cavodeassi et al., 2001, Kim et al., 2012, Li et al., 2014). IRX proteins all contain two highly conserved domains. The homeodomain is postulated to regulate interactions between transcriptional regulators by binding to genomic regions to regulate target gene expression, and the IRO Box is involved in protein-protein binding (Cavodeassi et al., 2001, Hiroi et al., 2001). Six IRX transcription factors have been identified (IRX1-IRX6), of which *Irx1*, *Irx2*, and *Irx4* cluster to chromosome 5 in humans (chromosome 13 in mice) and *Irx*3, *Irx*5, and *Irx6* cluster to chromosome 16 (chromosome 8 in mice) (Gaborit et al., 2012, Houweling et al., 2001). *Irx*3 and *Irx*5 expression is strikingly similar in developing mouse tissues (Houweling et al., 2001).

IRX proteins are required for the formation of limbs and skeletal tissues. *Irx1* is important for the specification of individual placodes through BMP signaling (Glavic et al., 2004). IRX3 has been shown to bind to the *Bmp10* promoter, which is important for ventricular septation, while IRX5 can bind to GATA3 and TRPS1 to regulate CXCL12 during bone progenitor migration in *Xenopus* embryos (Gaborit et al., 2012, Bonnard et al., 2012). *Irx1* and *Irx2* have been shown to regulate vertebrate digit formation, while *Irx*3 and *Irx*5 mediate early mouse limb bud specification by regulating *Gli*3 expression (Li et al., 2014, Becker et al., 2001, McDonald et al., 2010). Surprisingly, loss of *Irx*5 alone leads to a grossly normal mouse (Gaborit et al., 2012), while loss of both *Irx*3 and *Irx*5 together result in an embryonic lethal phenotype from cardiac defects and skeletal malformations (Li et al., 2014, Gaborit et al., 2012). Unfortunately, the early embryonic lethality of *Irx*3^−/−^/*Irx*5^−/−^ mice contributes to our incomplete understanding of the role of *Irx*3 and *Irx*5 in osteoblast function (Li et al., 2014, Gaborit et al., 2012).

Two mutations in human IRX5, Ala150Pro and Asn166Lys occur in patients with Hamamy syndrome (OMIM MIM611174; Bonnard et al., 2012), who present with craniofacial dysmorphism, osteopenia, tooth eruption defects, and hip dysplasia, along with cardiac defects and microcytic hypochromic anemia (Bonnard et al., 2012, Hamamy et al., 2007a, Hamamy et al., 2007b). The function of IRX5 seems to differ in mice and humans as the human phenotype is not observed in *Irx*5^−/−^ mice (Costantini et al., 2005). Iroquois homeodomains helix two and helix three are completely conserved in IRX1-IRX6 (Bonnard et al., 2012). The Hamamy mutations Ala150Pro and Asn166Lys occur in the IRX5 helix two or three respectively, indicating the importance of these residues in IRX homeodomain function. Irx5 was previously shown to form homodimers and heterodimers with Irx3 and Irx4 in transfected 10T1/2 cells (He et al., 2009). Previous meta-analysis of IRX3, IRX5, GATA3, and TRPS1 also indicated these proteins were involved in a regulatory network (Bonnard et al., 2012). Interestingly, patients with a 3.2 Mb deletion at 16q12.2–13, which includes *IRX*3, *IRX*5, and *IRX6*, show similar craniofacial features to those of Hamamy patients (Bonnard et al., 2012, Chang et al., 2010).

*Irx*3^−/−^ and *Irx*5^−/−^ mice do not show any Hamamy syndrome characteristics (Costantini et al., 2005, Smemo et al., 2014), but several aspects of the Hamamy syndrome phenotype are recapitulated in *Irx*3 and *Irx*5 double knockout mice (*Irx*3^−/−^/*Irx*5^−/−^ mice), including the cardiac defects and craniofacial dysmorphisms (Hiroi et al., 2001). Furthermore, the hind limb skeletal hypoplasia reported in *Irx*3^−/−^/*Irx*5^−/−^ mouse embryos and the femoral fragility observed in Hamamy syndrome patients suggest that IRX3 and IRX5 are important for limb development in vertebrates (Gaborit et al., 2012, Hamamy et al., 2007a). Although the *Irx*3^−/−^/*Irx*5^−/−^ mice have a complete loss of protein in comparison to the point mutations found in humans with Hamamy syndrome, the finding that multiple features of Hamamy syndrome phenotype are recapitulated in *Irx*3^−/−^/*Irx*5^−/−^ mice indicates that this mouse model may be useful for understanding the role of Iroquois proteins *in vivo*. Here, we avoid the embryonic lethality from cardiac malformations in *Irx*3^−/−^/*Irx*5^−/−^ mice by using the *Osx-Cre* transgene (Rodda & McMahon, 2006) to drive osteoblast-specific deletion of *Irx*3 in *Irx*5^−/−^ mice.

## 2 Results

### 2.1 Body size and craniofacial bone mineralization are reduced in newborn Irx3^flox/flox^/Irx5^−/−^/Osx-Cre^+^ mice

**Supplementary Fig. 1:**
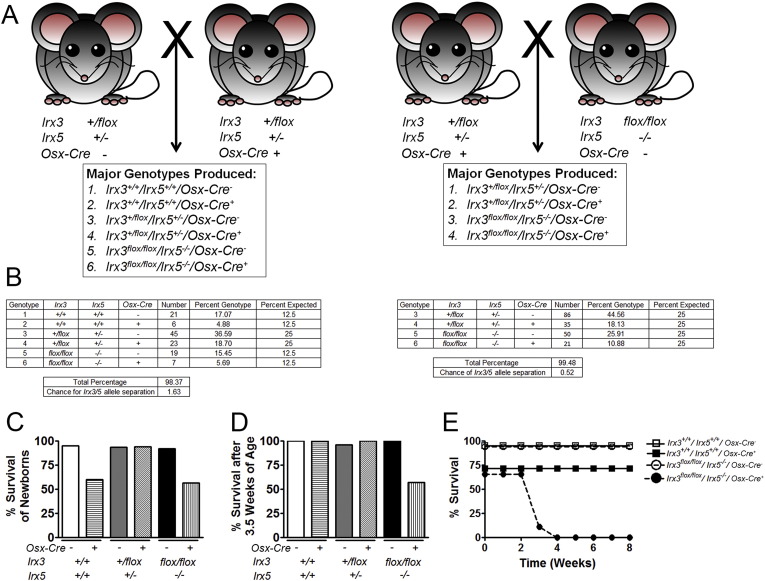
Mating strategies for the generation of *Irx*3^*flox*/*flox*^/*Irx*5^−/−^/*Osx-Cre*^+^ mice. (A) *Irx*3^*flox*/*flox*^/*Irx*5^−/−^/*Osx-Cre*^+^ mice and littermates used in this study were generated by crossing *Irx*3^+/*flox*^/*Irx*5^+/−^/*Osx-Cre*^−^ and *Irx*3^+/*flox*^/*Irx*5^+/−^/*Osx-Cre*^+^ mice (left) or *Irx*3^*flox*/*flox*^/*Irx*5^−/−^/*Osx-Cre*^−^ and *Irx*3^+/*flox*^/*Irx*5^+/−^/*Osx-Cre*^+^ mice (right). (B) Genotypes of newborn mice produced from the crossing of *Irx*3^+/*flox*^/*Irx*5^+/−^/*Osx-Cre*^−^ and *Irx*3^+/*flox*^/*Irx*5^+/−^/*Osx-Cre*^+^ mice (left) or *Irx*3^*flox*/*flox*^/*Irx*5^−/−^/*Osx-Cre*^−^ and *Irx*3^+/*flox*^/*Irx*5^+/−^/*Osx-Cre*^+^ mice (we also used *Irx*3^*flox*/*flox*^/*Irx*5^−/−^/*Osx-Cre*^−^ breeding to *Irx*3^+/*flox*^/*Irx*5^+/−^/*Osx-Cre*^+^ mice were used generate more *Irx*3^*flox*/*flox*^/*Irx*5^−/−^/*Osx-Cre*^+^ and control littermates. We noted no difference between single and double transgenic *Irx*3^*flox*/*flox*^/*Irx*5^−/−^/*Osx-Cre*^+^ mice but have omitted double transgenic mice from our analysis with the exception of the qPCR analysis. *Irx*3 and *Irx*5 allele separation events are approximately 0.5-2.0%. (C) Percent survival at birth of indicated genotypes from crossing *Irx*3^+/*flox*^/*Irx*5^+/−^/*Osx-Cre*^−^ and *Irx*3^+/*flox*^/*Irx*5^+/−^/*Osx-Cre*^+^ mice. Data is from n = 21 *Irx*3^+/+^/*Irx5*^+/+^/*Osx-Cre*^−^, n = 6 *Irx*3^+/+^/*Irx*5^+/+^/*Osx-Cre*^+^, *n* *=* *4*5 *Irx*3^+/*flox*^/*Irx*5^+/−^/*Osx-Cre*^−^, *n* *=* *2*3 *Irx*3^+/*flox*^/*Irx*5^+/−^/*Osx-Cre*^+^, *n* *=* *19 Irx*3^*flox*/*flox*^/*Irx*5^−/−^/*Osx-Cre*^−^, and *n* *=* *7 Irx*3^*flox*/*flox*^/*Irx*5^−/−^/*Osx-Cre*^+^. (D) Percentage of mice that survive at 3.5 weeks of age. Data is from n = 12 *Irx*3^+/+^/*Irx*5^+/+^/*Osx-Cre*^−^, n = 4 *Irx*3^+/+^/*Irx*5^+/+^/*Osx-Cre*^+^, *n* *=* *24 Irx*3^+/*flox*^/*Irx*5^+/−^/*Osx-Cre*^−^, *n* *=* *20 Irx*3^+/*flox*^/*Irx*5^+/−^/*Osx-Cre*^+^, *n* *=* 5 *Irx*3^*flox*/*flox*^/*Irx*5^−/−^/*Osx-Cre*^−^, and *n* *=* *4 Irx*3^*flox*/*flox*^/*Irx*5^−/−^/*Osx-Cre*^+^. (E) Kaplan-Meier survival curves of *Irx*3^*flox*/*flox*^/*Irx*5^−/−^/*Osx-Cre*^+^ mice compared to control genotypes. Data is from n = 40 *Irx*3^+/+^/*Irx*5^+/+^/*Osx-Cre*^−^, n = 28 *Irx*3^+/+^/*Irx*5^+/+^/*Osx-Cre*^+^, *n* *=* 5*1 Irx*3^*flox*/*flox*^/*Irx*5^−/−^/*Osx-Cre*^−^, and *n* *=* *29 Irx*3^*flox*/*flox*^/*Irx*5^−/−^/*Osx-Cre*^+^. Mice were omitted from the survival calculations if they were euthanized for experimental analysis.

**Supplementary Fig. 2:**
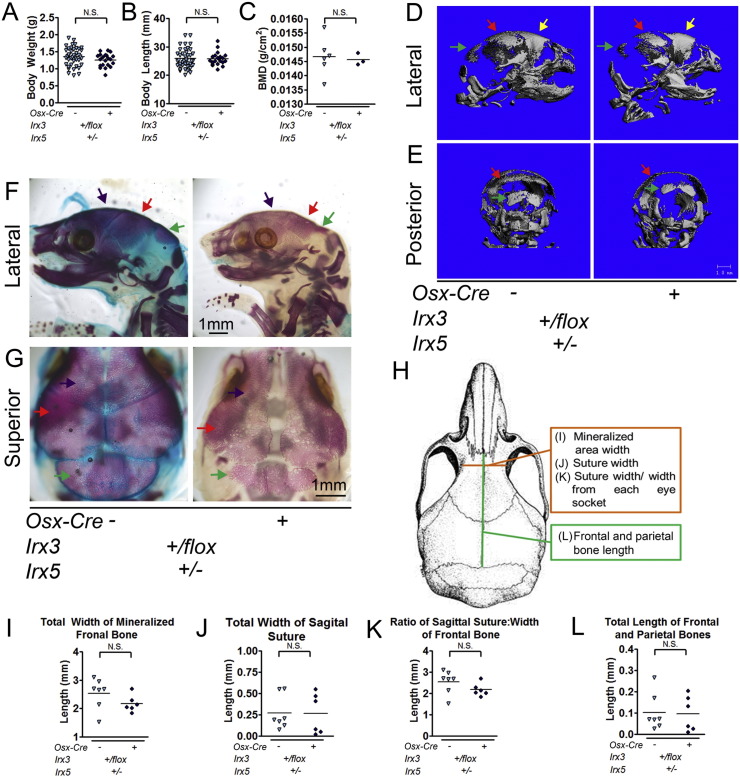
*Heterozygous Irx*3^+/*flox*^/*Irx*5^+/−^/*Osx-Cre*^+^ display reduced mineralization. (A) Newborn total body weight, (B) newborn body length, and (C) newborn bone mineral density (BMD) of lower right limb of indicated genotypes. Data are from n = 42 *Irx*3^+/*flox*^/*Irx*5^+/−^/*Osx-Cre*^−^ and n = 21 *Irx*3^+/*flox*^/*Irx*5^+/−^/*Osx-Cre*^+^. For BMD, n = 6 *Irx*3^+/*flox*^/*Irx*5^+/−^/*Osx-Cre*^−^ and n = 3 *Irx*3^+/*flox*^/*Irx*5^+/−^/*Osx-Cre*^+^. MicroCT imaging of newborn *Irx*3^+/*flox*^/*Irx*5^+/−^/*Osx-Cre*^−^ and *Irx*3^+/*flox*^/*Irx*5^+/*−*^/*Osx-Cre*^+^ mice. (D) Right lateral view of the skull. (E) Posterior view of the skull. Green arrows denote the interparietal bone, red arrows denote the parietal bones and yellow arrows denote the frontal bones. Data are representative of n = 2 of each genotype. (F) Superior view and (G) left lateral view of representative alizarin red and alcian blue staining of newborn *Irx*3^+/*flox*^/*Irx*5^+/−^/*Osx-Cre*^−^ and *Irx*3^+/*flox*^/*Irx*5^+/−^/*Osx-Cre*^+^ mice. Green arrows denote the interparietal bone, red arrows denote the parietal bones, and purple arrows denote the frontal bones. (H) Superior view images were measured for (I) the width of mineralized frontal bone, (J) sagittal suture width, (K) suture width divided by the width between the eye sockets, and (L) length of the frontal and parietal bones, measured along the sagittal suture. Photos and measurements are from n = 7 *Irx*3^+/*flox*^/*Irx*5^+/−^/*Osx-Cre*^−^ and n = 6 *Irx*3^+/*flox*^/*Irx*5^+/−^/*Osx-Cre*^+^. Statistical differences were determined by a Student’s t-test, *p < 0.05, **p < 0.01, ***p < 0.001. N.S., not significant.

**Fig. 1:**
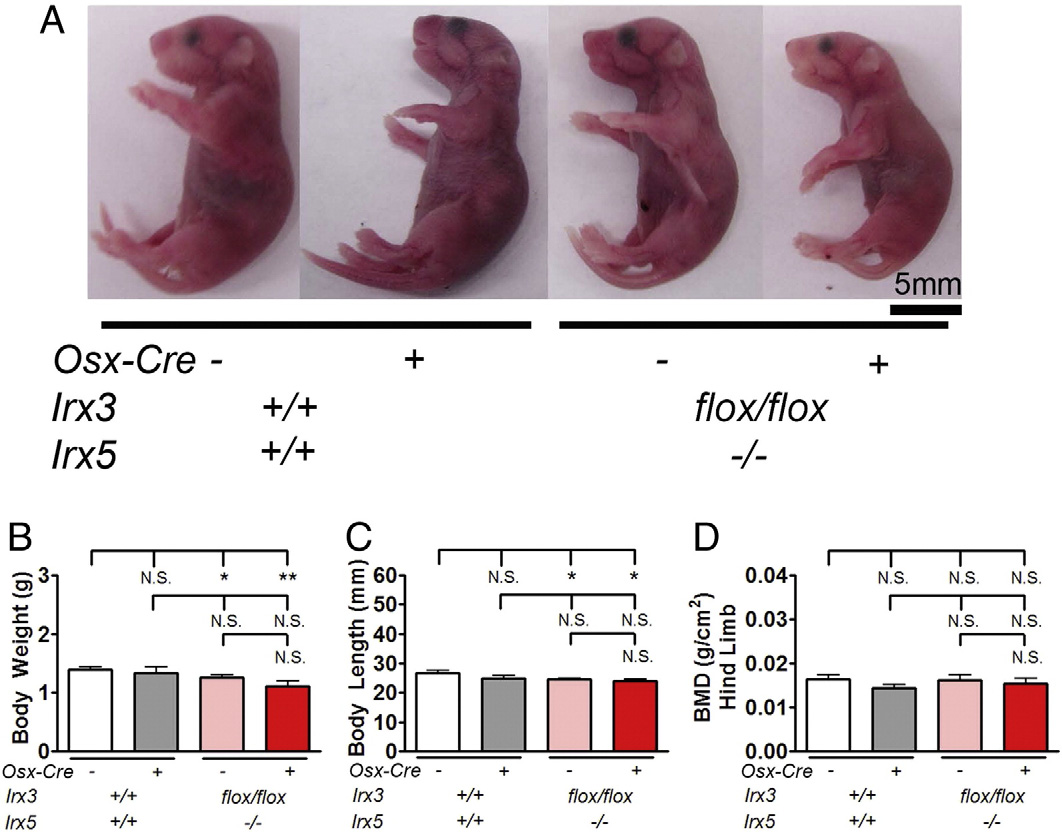
Osteoblastic specific deletion of *Irx*3 in newborn *Irx*5^−/−^ mice results in smaller mice. (A) Representative photos of newborn mice. (B) Newborn total body weight, (C) newborn body length, and (D) newborn BMD of lower right limb of indicated genotypes. Data are from *n* = 16 *Irx*3^+/+^/*Irx*5^+/+^/*Osx-Cre*^−^, *n* = 5 *Irx*3^+/+^/*Irx*5^+/+^/*Osx-Cre*^+^, *n* = 27 *Irx*3^*flox*/*flox*^/*Irx*5^−/−^/*Osx-Cre*^−^, *n* = 9 *Irx*3^*flox*/*flox*^/*Irx*5^−/−^/*Osx-Cre*^+^ for body weight and length measurements. For BMD, *n* = 3 *Irx*3^+/+^/*Irx*5^+/+^/*Osx-Cre*^−^, *n* = 3 *Irx*3^+/+^/*Irx*5^+/+^/*Osx-Cre*^+^, *n* = 3 *Irx*3^*flox*/*flox*^/*Irx*5^−/−^/*Osx-Cre*^−^, *n* = 3 *Irx*3^*flox*/*flox*^/*Irx*5^−/−^/*Osx-Cre*^+^. Statistical differences were determined by a Student's *t*-test, *p < 0.05, **p < 0.01, ***p < 0.001. N.S., not significant.

We focused our studies on newborn *Irx*3^*flox*/*flox*^/*Irx*5^−/−^/*Osx-Cre*^+^ mice (Supplementary Fig. 1A) to understand the role of *Irx*3 and *Irx*5 in early bone mineralization. The allelic separation of *Irx*3 and *Irx*5 was a rare event (approximately 0.5–2%, Supplementary Fig. 1B) and thus single allele mice were excluded from further analysis. Newborn *Irx*3^*flox*/*flox*^/*Irx*5^−/−^/*Osx-Cre*^+^ mice appeared grossly normal but slightly smaller than control littermates (Fig. 1A). 43% of *Irx*3^*flox/flox*^/*Irx*5^−/−^/*Osx-Cre*^+^ were viable at birth, which appeared to be an effect of the *Osx-Cre* allele as *Irx*3^+/+^/*Irx*5^+/+^/*Osx-Cre*^+^ also showed similar decreased neonatal viability (Supplementary Fig. 1C). However, *Irx*3^*flox/flox*^/*Irx*5^−/−^/*Osx-Cre*^+^ mice rarely survived to 4 weeks of age. This early lethality was not observed in 3–4-week-old *Irx*3^*flox/flox*^/*Irx*5^−/−^/*Osx-Cre*^−^ mice (Supplementary Fig. 1D) suggesting that the loss of Irx3 and Irx5 contributed to this decrease in survival in the neonatal stage. We observed small but statistically significant reductions in newborn total body weight and length in mice with a loss of *Irx*5 independent of *Osx-Cre* expression (Fig. 1B, C and Supplementary Fig. 2A and B). Lower limbs of *Irx*3^*flox*/*flox*^/*Irx*5^−/−^/*Osx-Cre*^+^ assessed by DEXA showed no differences in bone mineral density (Fig. 1D and Supplementary Fig. 2C). These data indicate that global deletion of *Irx*5 alone was sufficient to affect body size, but that additional deletion of *Irx*3 in osteoblastic cells did not have further effects on body size.

**Supplementary Fig. 3:**
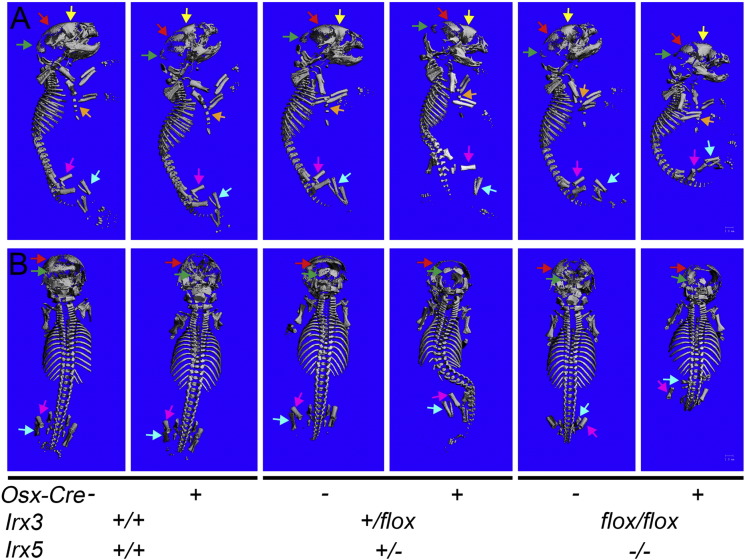
Whole body 3D microCT imaging of *Irx*3^*flox*/*flox*^/*Irx5*^−/−^/*Osx-Cre*^+^ mice and littermates. Representative whole body microCT imaging of newborn *Irx*3^*flox*/*flox*^/*Irx*5^−/−^/*Osx-Cre*^+^ mice and control littermates. (A) Right lateral view of the skeleton. (B) Posterior view of the skeleton. Data are representative n = 2 of each genotype. Green arrows denote the interparietal bone, red arrows denote the parietal bones, yellow arrows denote the frontal bones, orange arrows denote the sternum, pink arrows denote the femur, and blue arrows denote the tibia.

**Fig. 2:**
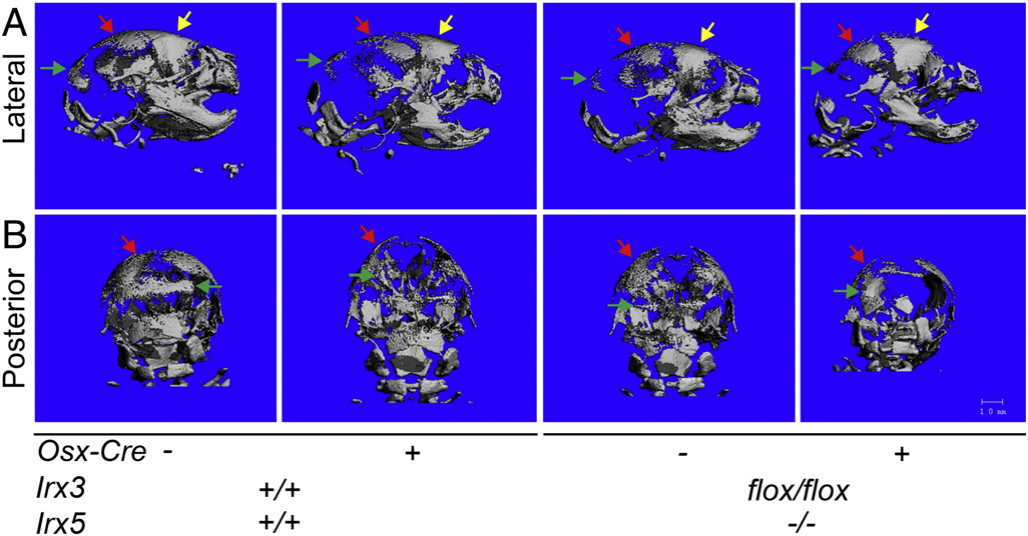
*Irx*3^*flox*/*flox*^/*Irx*5^−/−^/*Osx-Cre*^+^ mice have reduced skull mineralization. Representative skull microCT imaging of newborn *Irx*3^*flox*/*flox*^/*Irx*5^−/−^/*Osx-Cre*^+^ and control littermate skulls. (A) Right lateral view of the skull. (B) Posterior view of the skull. Green arrows denote the interparietal bone, red arrows denote the parietal bones, and yellow arrows denote the frontal bones. Images are representative of *n* = 2 of each genotype. (For interpretation of the references to color in this figure legend, the reader is referred to the web version of this article.)

During the course of our studies, it became evident that *Irx*3^+/+^/*Irx*5^+/+^/*Osx-Cre*^+^ mice had an unexpected basal phenotype with reductions in the mineralized area of the frontal bone. This was confirmed by recent reports of effects by the *Osx-Cre* transgene alone on craniofacial mineralization (Wang et al., 2015, Huang and Olsen, 2015). MicroCT 3D image reconstruction showed both *Irx*3^+/*flox*^/*Irx*5^+/−^/*Osx-Cre*^+^ and *Irx*3^*flox*/*flox*^/*Irx*5^−/−^/*Osx-Cre*^+^ mice had reduced mineralization in the frontal, parietal, and interparietal bones (Fig. 2 and Supplementary Figs. 2D, E, and 3). The long bones from both *Irx*3^+/*flox*^/*Irx*5^+/−^/*Osx-Cre*^+^ and *Irx*3^*flox*/*flox*^/*Irx*5^−/−^/*Osx-Cre*^+^ newborn mice also appeared unaffected by microCT.

**Fig. 3:**
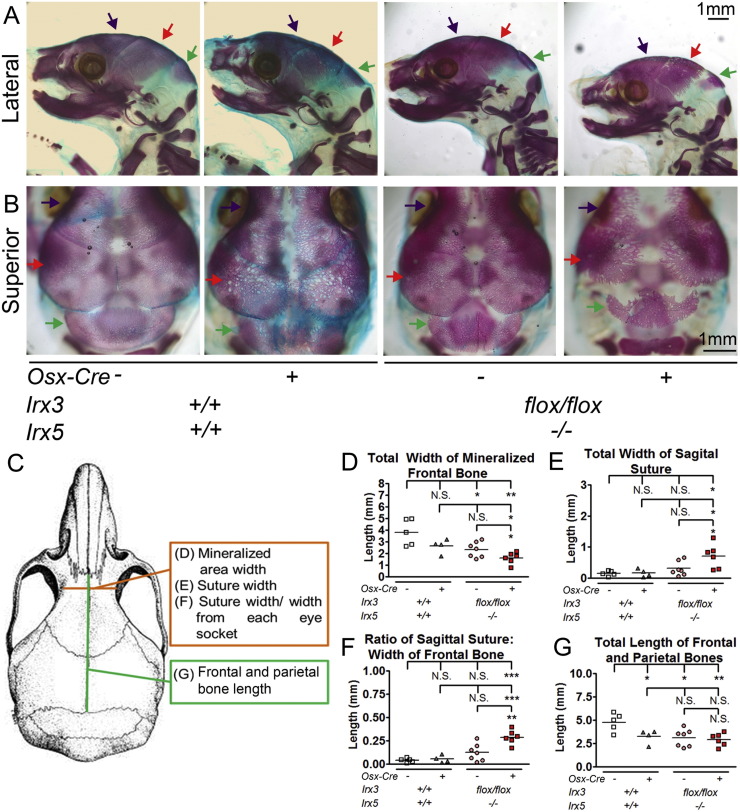
*Irx*3^*flox*/*flox*^/*Irx*5^−/−^/*Osx-Cre*^+^ display reduced mineralization in frontal, parietal, and interparietal bones. (A) Left lateral view and (B) superior view of representative alizarin red and alcian blue stained newborn mice. Green arrows denote the interparietal bone, red arrows denote the parietal bones, and purple arrows denote the frontal bones. (C) Superior view images were measured for (D) the width of mineralized frontal bone, (E) sagittal suture width, (F) suture width divided by the width between the eye sockets, and (G) length of the frontal and parietal bones, measured along the sagittal suture. Photos and measurements are from *n* = 5 *Irx*3^+/+^/*Irx*5^+/+^/*Osx-Cre*^−^, *n* = 4 *Irx*3^+/+^/*Irx*5^+/+^/*Osx-Cre*^+^, *n* = 7 *Irx*3^*flox*/*flox*^/*Irx*5^−/−^/*Osx-Cre*^−^, *n* = 6 *Irx*3^*flox*/*flox*^/*Irx*5^−/−^/*Osx-Cre*^+^. Statistical differences were determined by a Student's *t*-test, *p < 0.05, **p < 0.01, ***p < 0.001. N.S., not significant. (For interpretation of the references to color in this figure legend, the reader is referred to the web version of this article.)

We next performed whole body skeletal staining of newborn *Irx*3^*flox*/*flox*^/*Irx*5^−/−^/*Osx-Cre*^+^ mice with alizarin red and alcian blue. *Irx*3^+/*flox*^/*Irx*5^+/−^/*Osx-Cre*^+^ and *Irx*3^*flox*/*flox*^/*Irx*5^−/−^/*Osx-Cre*^+^ mice showed reduced mineralization in frontal, parietal, and interparietal bones (Fig. 3A and 3B). We measured the frontal bone total mineralized area, using the orbits as landmarks; the suture width in the same area; the length of the parietal and frontal bones along the suture; and the interparietal bone width (Fig. 3C). As noted above, we found reductions in the mineralized area of the frontal bone in *Irx*3^+/+^/*Irx*5^+/+^/*Osx-Cre*^+^ mice; however, *Irx*3^+/*flox*^/*Irx*5^+/−^/*Osx-Cre*^+^ and *Irx*3^*flox*/*flox*^/*Irx*5^−/−^/*Osx-Cre*^+^ mice showed an even greater reduction in frontal bone mineralization (Fig. 3D and Supplementary Fig. 2F-I). Additionally, the width of the suture was significantly greater in *Irx*3^*flox*/*flox*^/*Irx*5^−/−^/*t-Cre*^−^ mice than in *Irx*3^+/+^/*Irx*5^+/+^/*Osx-Cre*^+^ and wildtype mice (Fig. 3E and F). The parietal and frontal bone total length showed a reduction caused by the *Osx-Cre* but not with *Irx*3 or *Irx*5 loss (Fig. 3G). We noted no significant differences between *Irx*3^+/*flox*^/*Irx*5^+/−^/*Osx-Cre*^−^ and *Irx*3^+/*flox*^/*Irx*5^+/−^/*Osx-Cre*^+^ mouse cranial measurements (Supplementary Fig. 2F-L). These data indicate that the presence of the *Osx-Cre* transgene alone can reduce cranial mineralization, but the absence of *Irx*3 and *Irx*5 in osteoblastic cells leads to a greater reduction in bone mineralization.

### 2.2 Irx3^flox/flox^/Irx5^−/−^/Osx-Cre^+^ skulls have reduced osteoblastic mineralization

**Fig. 4:**
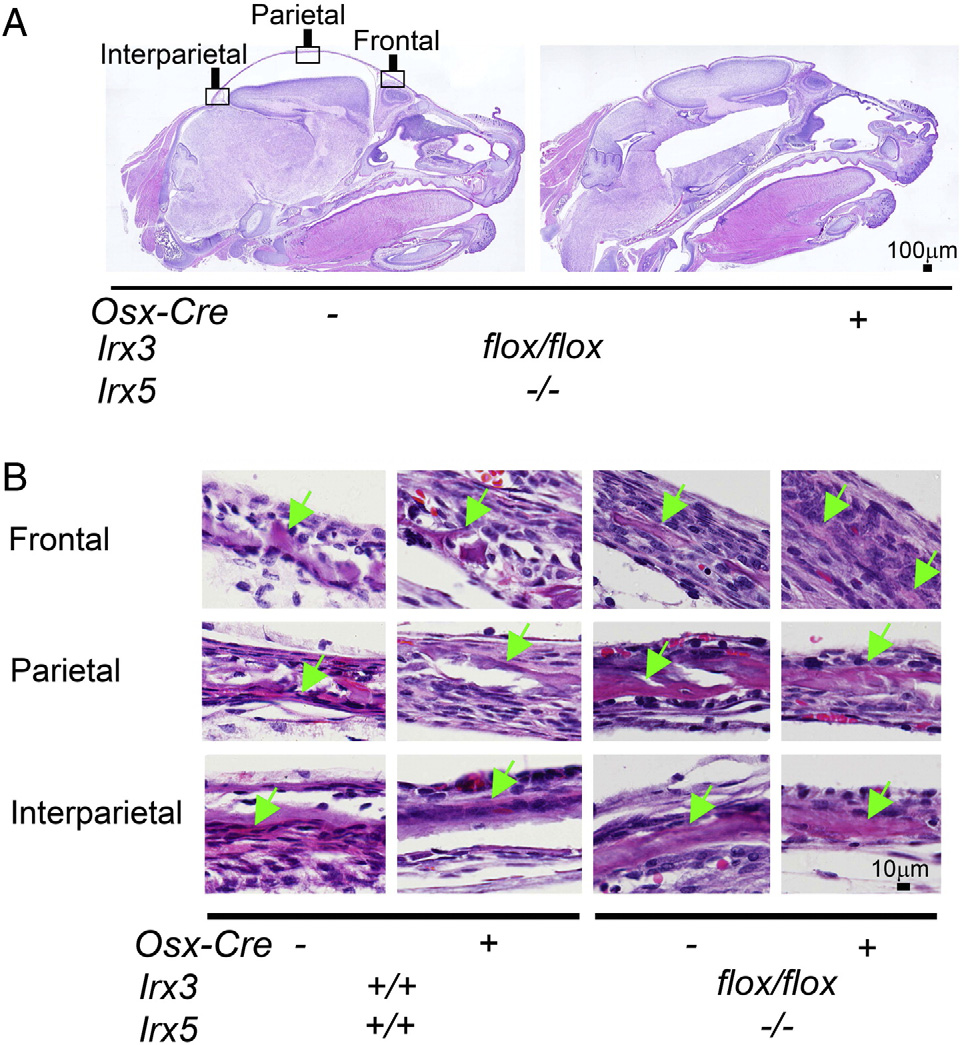
Osteoblastic bone mineralization is reduced in *Irx*3^*flox*/*flox*^/*Irx*5^−/−^/*Osx-Cre*^+^ skulls. (A) Representative sagittal sections of *Irx*3^*flox*/*flox*^/*Irx*5^−/−^/*Osx-Cre*^−^*and Irx*3^*flox*/*flox*^/*Irx*5^−/−^/*Osx-Cre*^+^ skulls stained with hematoxylin and eosin. Frontal, parietal, and interparietal bones are shown in the boxes and taken at higher magnification to observe cellular morphology. (B) *Irx*3^+/+^/*Irx*5^+/+^/*Osx-Cre*^−^, *Irx*3^+/+^/*Irx*5^+/+^/*Osx-Cre*^+^, *Irx*3^*flox*/*flox*^/*Irx*5^−/−^/*Osx-Cre*^−^, *and Irx*3^*flox*/*flox*^/*Irx*5^−/−^/*Osx-Cre*^+^ enlarged photos of frontal, parietal, interparietal, and occipital bones. Green arrows denote regions with mineralized bone in *Irx*3^*flox*/*flox*^/*Irx*5^−/−^/*Osx-Cre*^+^ mice and control littermates. Data is representative *n* = 3 *Irx*3^+/+^/*Irx*5^+/+^/*Osx-Cre*^−^, *n* = 3 *Irx*3^+/+^/*Irx*5^+/+^/*Osx-Cre*^+^, *n* = 5 *Irx*3^*flox*/*flox*^/*Irx*5^−/−^/*Osx-Cre*^−^, *n* = 4 *Irx*3^*flox*/*flox*^/*Irx*5^−/−^/*Osx-Cre*^+^. (For interpretation of the references to color in this figure legend, the reader is referred to the web version of this article.)

We next used hematoxylin and eosin staining to identify alterations to the bone architecture and mineralization in *Irx*3^*flox*/*flox*^/*Irx*5^−/−^/*Osx-Cre*^+^ mice. We found that bone accumulation was less in *Irx*3^*flox*/*flox*^/*Irx*5^−/−^/*Osx-Cre*^+^ mice in the parietal and interparietal bones than in *Irx*3^*flox*/*flox*^/*Irx*5^−/−^/*Osx-Cre*^−^ mice (Fig. 4A). Closer inspection of the frontal, parietal, and interparietal bones revealed a reduction in the thickness of mineralized bone in *Irx*3^*flox*/*flox*^/*Irx*5^−/−^/*Osx-Cre*^+^ mice with no major change in cuboidal osteoblasts adjacent to bony surfaces (Fig. 4B, green arrows). Occipital bones showed no significant differences among the genotypes (data not shown). These histological analyses suggest that there is not a major change in osteoblasts morphology and numbers at the bone surfaces, but decreased osteoblast mineralization caused by loss of *Irx3* and *Irx*5.

### 2.3 Irx3^flox/flox^/Irx5^−/−^/Osx-Cre^+^ skulls have reduced expression of genes that regulate osteoblastic mineralization

**Fig. 5:**
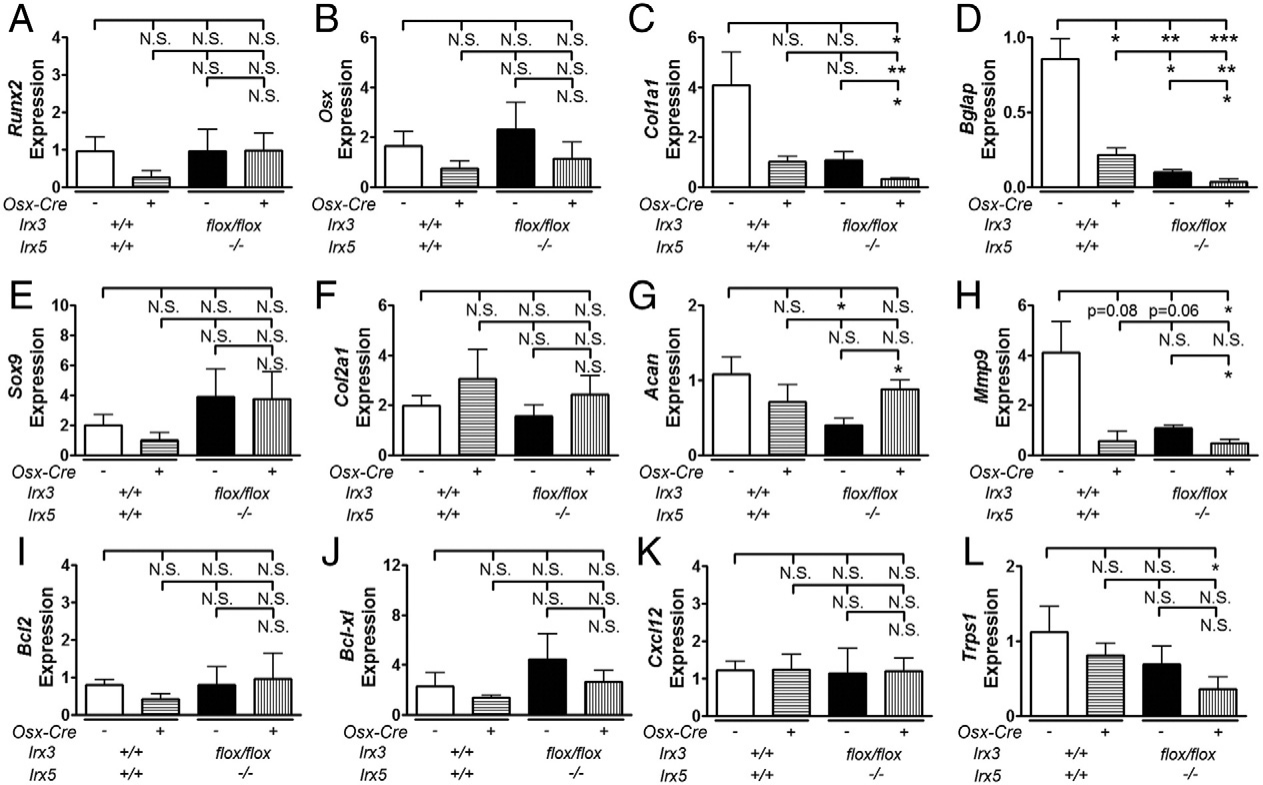
Mineralization and chondrocyte gene expression is reduced expression in *Irx*3^*flox*/*flox*^/*Irx*5^−/−^/*Osx-Cre*^+^ calvaria. Relative expression levels of osteoblastic genes (A) *Runx2*, (B) *Osx*, (C) *Col1a1*, and (D) *Bglap*. Relative expression levels of chondrogenic genes (E) *Sox9*, (F) *Col2a1*, (G) *Acan*, and (H) *Mmp9*. Relative expression levels of apoptosis related genes (I) *Bcl2 and* (J) *Bcl-xl*. Relative expression levels of *Irx*3 and *Irx*5 related genes (K) *Cxcl12* and (L) *Trps1*. All samples are from whole newborn calvaria, *n* = 5 *Irx*3^+/+^/*Irx*5^+/+^/*Osx-Cre*^−^, *n* = 3 *Irx*3^+/+^/*Irx*5^+/+^/*Osx-Cre*^+^, *n* = 4 *Irx*3^*flox*/*flox*^/*Irx*5^−/−^/*Osx-Cre*^−^, *n* = 5 *Irx*3^*flox*/*flox*^/*Irx*5^−/−^/*Osx-Cre*^+^. Whole calvaria samples were normalized to *Irx*3^+/+^/*Irx*5^+/+^/*Osx-Cre*^−^ mice. Statistical differences were determined by a Student's *t*-test, *p < 0.05, **p < 0.01, ***p < 0.001. N.S., not significant.

We next examined whole calvarial expression of genes involved in osteoblast mineralization and maturation. Early markers of osteoblast lineage specification such as *Runx2* (Fig. 5A) and *Osx* (Fig. 5B) were not significantly altered; however, the mature osteoblast markers *Col1a1* (Fig. 5C) and *Osteocalcin* (*Bglap*) (Fig. 5D) were significantly reduced in *Irx*3^*flox*/*flox*^/*Irx*5^−/−^/*Osx-Cre*^+^ calvaria, even in relation to the reductions in *Bglap* expression in *Irx*3^+/+^/*Irx*5^+/+^/*Osx-Cre*^+^ and *Irx*3^*flox*/*flox*^/*Irx*5^−/−^/*Osx-Cre*^−^ control calvaria. *Enam* and *Tifip11*, two genes implicated in mineralization, were not changed in expression in *Irx*3^*flox*/*flox*^/*Irx*5^−/−^/*Osx-Cre*^+^ calvaria (data not shown).

We next examined calvarial gene expression of chondrogenesis using *Sox9*, *Col2a1*, *Acan*, and *Mmp9*, since mesenchymal cells from intermembranous bones maintain chondrogenic gene expression (Aberg et al., 2005). There were no observable differences in *Sox9* (Fig. 5E) and *Col2a1* (Fig. 5F) levels, although we observed significant reductions of *Acan* (Fig. 5G) in *Irx*3^*flox*/*flox*^/*Irx*5^−/−^/*Osx-Cre*^−^ calvaria that were not present in *Irx*3^*flox*/*flox*^/*Irx*5^−/−^/*Osx-Cre*^+^ calvaria. We observed a significant reduction in *Mmp9* expression in *Irx*3^*flox*/*flox*^/*Irx*5^−/−^/*Osx-Cre*^+^ calvaria that was not observed in the other genotypes, even though *Irx*3^+/+^/*Irx*5^+/+^/*Osx-Cre*^+^ and *Irx*3^*flox*/*flox*^/*Irx*5^−/−^/*Osx-Cre*^−^ calvaria were modestly reduced in *Mmp9* expression (Fig. 5H). We observed no differences in expression of apoptosis related genes *Bcl2* and *Bcl-xl* in *Irx*3^*flox*/*flox*^/*Irx*5^−/−^/*Osx-Cre*^+^ calvaria (Fig. 5I-J). This indicated that loss of *Irx*3 and *Irx*5 together in osteoblastic lineage cells affects later osteogenic genes more significantly than chondrocyte genes and does not result in increased apoptosis of osteogenic cells in the calvaria.

In studies using *Xenopus laevis* embryos, IRX5 interacted with GATA3 and TRPS1, forming a complex that down regulated CXCL12 production (Bonnard et al., 2012), although IRX3 and IRX5 did not directly influence *Gata*3 transcription (data not shown). We examined the expression of *Cxcl12* and *Trps1* in order to determine if the reduced bone mineralization in *Irx*3^*flox*/*flox*^/*Irx*5^−/−^/*Osx-Cre*^+^ mice was through downstream mediators of *Gata*3, *Irx*3, and *Irx*5. *Cxcl12* expression was not significantly altered in *Irx*3^*flox*/*flox*^/*Irx*5^−/−^/*Osx-Cre*^+^ mice (Fig. 5K). Interestingly, there was a significant reduction in *Trps1* in *Irx*3^*flox*/*flox*^/*Irx*5^−/−^/*Osx-Cre*^+^ calvaria (Fig. 5L), a gene that can reduce *Bglap* expression *in vitro* and is required for proper osteoblast mineralization (Piscopo et al., 2009, Kuzynski et al., 2014). These findings suggest a role for *Irx*3 and *Irx*5 in the regulation osteoblast mineralization gene expression and suggest that in mineralization, *Irx*3 and *Irx*5 may function through a pathway that is distinct from *Gata*3.

### 2.4 Older Irx3^flox/flox^/Irx5^−/−^/Osx-Cre^+^ mice have reduced bone mineralization

**Fig. 6:**
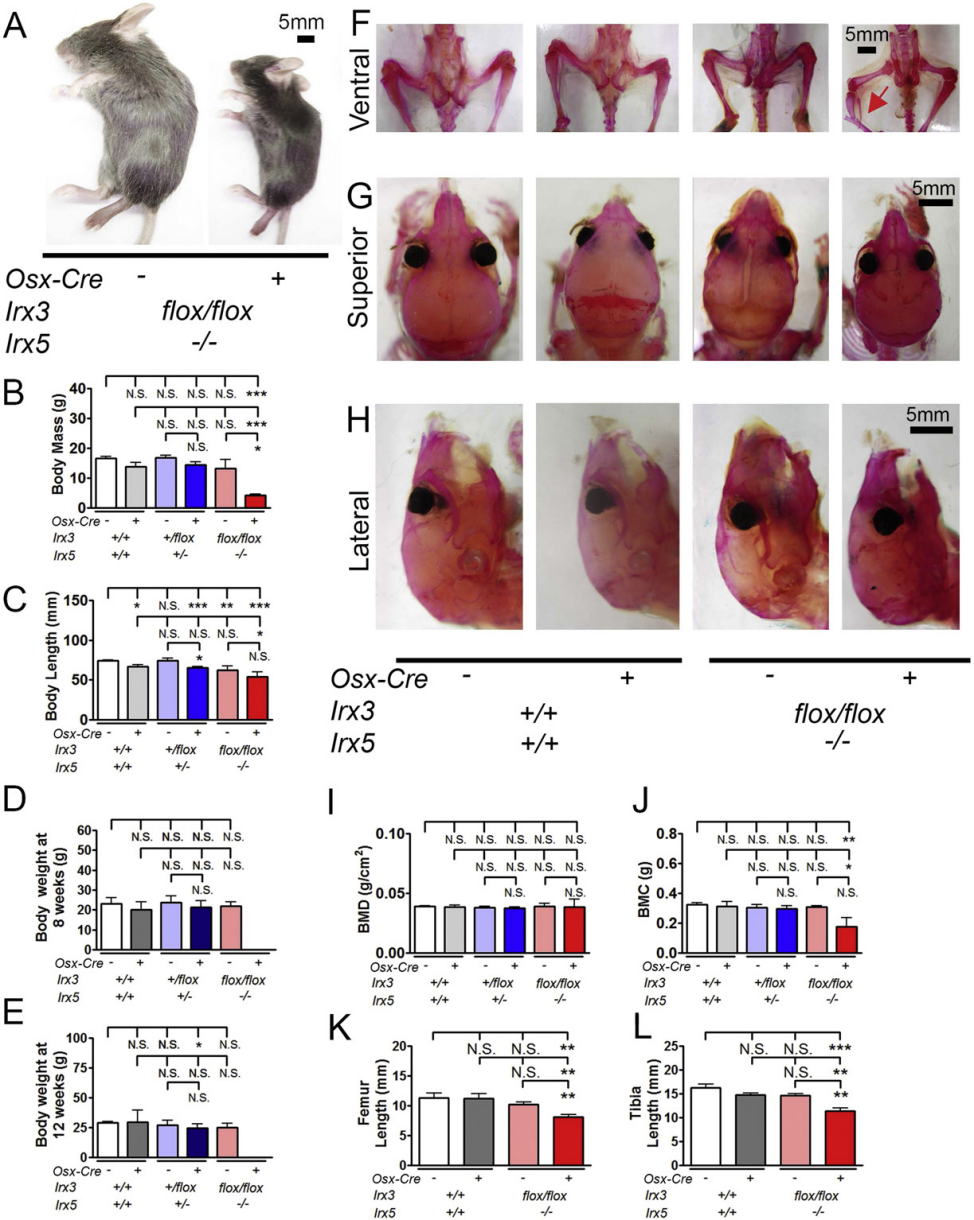
Postnatal *Irx*3^*flox*/*flox*^/*Irx*5^−/−^/*Osx-Cre*^+^ mice show reductions in body weight and body length. (A) Representative photos of *Irx*3 and *Irx*5 genotypes *Irx*3^*flox*/*flox*^/*Irx*5^−/−^/*Osx-Cre*^−^, *and Irx*3^*flox*/*flox*^/*Irx*5^−/−^/*Osx-Cre*^+^ mice at 3.5 weeks of age. (B) Body weight and (C) body length of 3–4-week-old *Irx*3^*flox*/*flox*^/*Irx*5^−/−^/*Osx-Cre*^+^ mice and littermate controls. Body weight of (D) 8- and (E) 12-week-old mouse littermate controls, without *Irx*3^*flox*/*flox*^/*Irx*5^−/−^/*Osx-Cre*^+^ mice due to lethality at 4 weeks of age. (F) Ventral, (G) superior, and (H) lateral views of alizarin red and alcian blue stained *Irx*3^*flox*/*flox*^/*Irx*5^−/−^/*Osx-Cre*^+^ and control littermates. Red arrow in (F) denotes spontaneous fracture in *Irx*3^*flox*/*flox*^/*Irx*5^−/−^/*Osx-Cre*^+^ tibia. 3–4-week old whole body (I) bone mineral density (BMD) and (J) bone mineral content (BMC) of *Irx*3^*flox*/*flox*^/*Irx5*^−/−^/*Osx-Cre*^+^ and control littermates. Femur (K) and tibia (L) length from 3 to 4 week old *Irx*3^*flox*/*flox*^/*Irx*5^−/−^/*Osx-Cre*^+^ and control littermates. 3–4-week old body weight and length old data is from *n* = 15 (*n* = 10 for length measurement) *Irx*3^+/+^/*Irx*5^+/+^/*Osx-Cre*^−^, *n* = 10 (*n* = 9 for length measurement) *Irx*3^+/+^/*Irx*5^+/+^/*Osx-Cre*^+^, *n* = 15 (*n* = 12 for length measurement) *Irx*3^+/*flox*^/*Irx*5^+/*−*^/*Osx-Cre*^−^, *n* = 12 (*n* = 13 for length measurement) *Irx*3^+/*flox*^/*Irx*5^+/−^/*Osx-Cre*^+^, *n* = 3 (*n* = 3 for length measurement) *Irx*3^*flox*/*flox*^/*Irx*5^−/−^/*Osx-Cre*^−^, *and n* = 4 (*n* = 4 for length measurement) *Irx*3^*flox*/*flox*^/*Irx*5^−/−^/*Osx-Cre*^+^ mice. For body mass measurements at 8 and 12 weeks of age, data are from *n* = 7 *Irx*3^+/+^/*Irx*5^+/+^/*Osx-Cre*^−^, *n* = 5 *Irx*3^+/+^/*Irx*5^+/+^/*Osx-Cre*^+^, *n* = 10 *Irx*3^+/*flox*^/*Irx*5^+/−^/*Osx-Cre*^−^, *n* = 7 *Irx*3^+/*flox*^/*Irx*5^+/−^/*Osx-Cre*^+^, *and n* = 2 *Irx*3^*flox*/*flox*^/*Irx*5^−/−^/*Osx-Cre*^−^ mice, pooled sexes. For BMD and BMC, data are from *n* = 8 *Irx*3^+/+^/*Irx*5^+/+^/*Osx-Cre*^−^, *n* = 5 *Irx*3^+/+^/*Irx*5^+/+^/*Osx-Cre*^+^, *n* = 9 *Irx*3^+/*flox*^/*Irx*5^+/−^/*Osx-Cre*^−^, *n* = 8 *Irx*3^+/*flox*^/*Irx*5^+/−^/*Osx-Cre*^+^, *n* = 2 *Irx*3^*flox*/*flox*^/*Irx*5^−/−^/*Osx-Cre*^−^ mice, and *n* = 2 *Irx*3^*flox*/*flox*^/*Irx*5^−/−^/*Osx-Cre*^+^ mice, pooled sexes. For femur and tibia measurements, data are from *n* = 6 (*n* = 5 for tibia) *Irx*3^+/+^/*Irx*5^+/+^/*Osx-Cre*^−^, *n* = 6 *Irx*3^+/+^/*Irx*5^+/+^/*Osx-Cre*^+^, *n* = 5 *Irx*3^*flox*/*flox*^/*Irx*5^−/−^/*Osx-Cre*^−^ mice, and *n* = 11 *Irx*3^*flox*/*flox*^/*Irx*5^−/−^/*Osx-Cre*^+^ mice, pooled sexes. Statistical differences were determined by a Student's *t*-test, *p < 0.05, **p < 0.01, ***p < 0.001. N.S., not significant.

We next looked at whole body and skeletal mineralization of *Irx*3^*flox*/*flox*^/*Irx*5^−/−^/*Osx-Cre*^+^ mice that survived to 3–4 weeks of age to identify if there were similar reductions in bone mineralization described in Hammy patients (Hamamy et al., 2007a). 3–4-week-old *Irx*3^*flox/flox*^/*Irx*5^−/−^/*Osx-Cre*^+^ mice had significant reductions in body size and length (Fig. 6A-C), while body weights remained comparable in all the other genotypes through 12 weeks of age, with the exception of a significant decrease in bodyweight in 12 week old *Irx*3^+/*flox*^/*Irx*5^+/−^/*Osx-Cre*^+^ mice (Fig. 6D-E). Alizarin red and alcian blue staining of 3–4-week-old *Irx*3^*flox*/*flox*^/*Irx*5^−/−^/*Osx-Cre*^+^ mice revealed an overall reduction bone mineralization with gross skeletal abnormalities and spontaneous fractures in 3 out of the 10 mice that were analyzed (Fig. 6F). Additionally, cranial bone mineralization appeared reduced in *Irx*3^*flox*/*flox*^/*Irx*5^−/−^/*Osx-Cre*^+^ mice, mineralized by 3.5 weeks of age (Supplementary Fig. 6G-H). *Irx*3^*flox*/*flox*^/*Irx*5^−/−^/*Osx-Cre*^+^ mice whole body bone mineral density (BMD) were unchanged compared to control littermates but there was a significant decrease in bone mineral content (BMC), consistent with the reduced bone size (Supplementary Fig. 6I-L). Unfortunately, the high lethality of *Irx*3^*flox/flox*^/*Irx*5^−/−^/*Osx-Cre*^+^ mice limited our ability to generate sufficient numbers to adequately analyze the 4 week phenotype further. These data indicate that deletion of *Irx*3 and *Irx*5 in osteoblastic cells can influence both body size and bone mineralization in both newborn and older mice.

### 2.5 Bone density is reduced in Hamamy syndrome patients

Hamamy patient mutations in IRX5 result in craniofacial dysmorphisms and mineralization defects while loss of *Irx*5 in mice results in no detectable bone abnormalities (Li et al., 2014). Since the global loss of both *Irx*3 and *Irx*5 leads to cardiac phenotypes similar to those seen in Hamamy patients, we wanted to see if the decreased mineralization we found in *Irx*3^*flox*/*flox*^/*Irx*5^−/−^/*Osx-Cre*^+^ mice also reflected the clinical presentation of Hamamy syndrome patients.

Patients with Hamamy syndrome at 8 and 9 years of age displayed reduced bone mineral density with spine lumbar *Z*-scores of − 3.7 and − 1.5 (Table 1). Bone mineral density improved with age in these patients to − 2.7 and − 1.4 at ages 19 and 20 years of age, respectively (Table 1). The femoral *Z*-score was determined at 9 years of age for one Hamamy patient (femoral Z-score of − 2.2), but both patients femoral Z-scores remained above − 1.0 at 19 and 20 years of age (Table 1). We noted dramatic reductions in bone mineral content in 3–4 week old *Irx*3^*flox/flox*^/*Irx*5^−/−^/*Osx-Cre*^+^ mice with spontaneous fractures in 3 out of 10 mice, indicating that 3–4 week old *Irx*3^*flox/flox*^/*Irx*5^−/−^/*Osx-Cre*^+^ mice have similarities to Hamamy patient bone mineralization. Furthermore, the spontaneous fractures observed in 3–4 week old *Irx*3^*flox/flox*^/*Irx*5^−/−^/*Osx-Cre*^+^ mice resembles the bone fragility reported in femora and other long bones of 8–10 year old Hamamy patients (Hamamy et al., 2007a).

## 3 Discussion

**Fig. 7:**
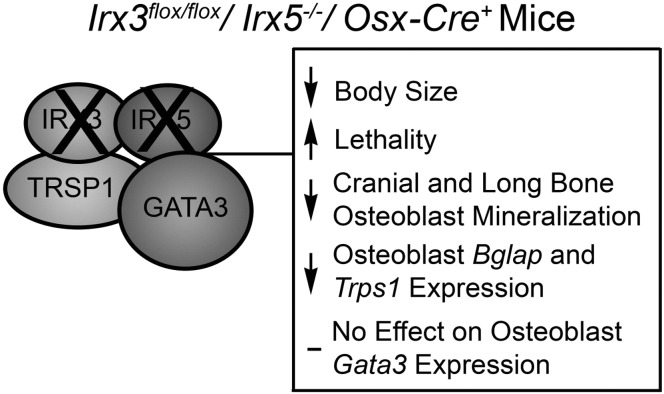
Model for the effect of Irx3 and Irx5 on bone mineralization. IRX3 and IRX5 are required for proximal and anterior limb development and maintain a role in osteoblast mineralization. In the absence of *Irx*3 and *Irx*5 in osteoblast lineage cells, there is a reduction in osteoblast mineralization, most notably in bones that undergo intramembranous ossification and later in the general skeleton. Osteoblastic cells that lack IRX3 and IRX5 display reduced *Col1a1*, *Bglap*, *Mmp9*, and *Trps1* and there is reduced bone formation in intramembranous bones, specifically in the frontal, parietal, and interparietal bones.

Proper craniofacial development requires control of bone mineralization by osteoblastic cell lineages (Mackie et al., 2008, Percival and Richtsmeier, 2013). Our studies show that osteoblast-specific loss of *Irx*3 and *Irx*5 leads to impaired mineralization in a very specific subset of cranial bones, possibly by blocking their expression of mature osteoblast mineralization genes (Fig. 7).

During the course of our study, we unexpectedly discovered that *Osx-Cre* mice have a newborn mineralization defect independent of the *Irx*3 and *Irx*5 mutation status. Our studies are consistent with recent reports that *Osx-Cre* mice alone have a newborn bone mineralization defect, specifically in intramembranous bones (Wang et al., 2015, Huang and Olsen, 2015). Interestingly, the absence of both *Irx*3 and *Irx*5 in osteoblastic cells caused an even more dramatic defect in intramembranous mineralization. Furthermore, *Osx-Cre* mice can survive past weaning and later stages of bone development occur normally, whereas *Irx*3^*flox*/*flox*^/*Irx*5^−/−^/*Osx-Cre*^+^ mice experience premature lethality around 3.5–4 weeks of age with bone fragility and spontaneous fractures. This indicates that the absence of both *Irx*3 and *Irx*5 in osteoblastic cells can influence neonatal survival at later stages of development. Our findings also emphasize the importance of using *Osx-Cre*^+^ littermates as controls for studies involving skeletal development. Furthermore, our results suggest that other Cre drivers, such as *Runx2-Cre* or *Bglap-Cre* mice, may be useful for future studies to confirm early skeletal mineralization phenotypes (Elefteriou & Yang, 2011).

*Irx*3^*flox*/*flox*^/*Irx*5^−/−^/*Osx-Cre*^+^ mice that survive to 3–4 weeks of age have smaller femora and tibiae and appeared to have signs of bone fragility, which is consistent with reports of Hamamy syndrome patients developing bone fragility and long bone fractures later in life (Hamamy et al., 2007a). Hamamy syndrome patients also had reduced BMD that was not observed in either newborn or 3–4 week old *Irx*3^*flox*/*flox*^/*Irx*5^−/−^/*Osx-Cre*^+^ mice. BMD measurements in mice are not particularly sensitive and more detailed analysis of *Irx*3^*flox*/*flox*^/*Irx*5^−/−^/*Osx-Cre*^+^ bones may be warranted. Our data demonstrate that IRX3 and IRX5 are important for both early osteoblast mineralization function and later skeletal mineralization, which also will help in understanding the bone fragility that occurs in Hamamy patients.

Our use of osteoblast specific deletion of *Irx*3 in *Irx*5^−/−^ mice differs from previous models that have germ-line deletions of *Irx*3 and *Irx*5 or deletions of *Irx1* and *Irx2* in chick embryos, all of which showed severe limb defects (Li et al., 2014, Diaz-Hernandez et al., 2013). We were surprised to find that use of the *Osx-Cre* to delete *Irx*3 in *Irx*5^−/−^ mice did not lead to significant limb malformations in newborn *Irx*3^*flox*/*flox*^/*Irx*5^−/−^/*Osx-Cre*^+^ mice; this is likely due to the fact that endochondral bone patterning is determined much earlier in development and may involve a different subset of cell types (Mackie et al., 2008, Knothe Tate et al., 2008). Furthermore, *Irx*3 and *Irx*5 germline deletion resulted in increased sonic hedgehog signaling sensitivity through upregulation of *Ptc1* and *Gli1*, which are important for early establishment of limb formation and osteoblast proliferation, but the contribution to the expression of bone mineralization genes was minimal (Li et al., 2014, Pan et al., 2013). This further supports the notion that *Irx*3 and *Irx*5 play roles in both limb and cranial development, and in cranial and limb mineralization by osteoblast lineage cells.

While it is clear that IRX3 and IRX5 can regulate cranial bone mineralization, the mechanism for how IRX3 and IRX5 control bone mineralization remains unclear. IRX5 might bind to GATA3 and TRPS1 proteins in a complex that regulates cranial neural crest cell migration (Bonnard et al., 2012). However, our results did not show differential expression of *Cxcl12* (Gaborit et al., 2012), a target of IRX3 and IRX5 which is thought to be regulated by GATA3. In addition, the affected bones in *Irx*3^*flox*/*flox*^/*Irx*5^−/−^/*Osx-Cre*^+^ mice were largely derived from the mesenchyme, rather than neural crest derivatives. Furthermore, *Trps1* levels were significantly reduced in *Irx*3^*flox*/*flox*^/*Irx*5^−/−^/*Osx-Cre*^+^ mice suggesting that loss of mineralization is occurring through a *Trps1* specific pathway (Piscopo et al., 2009). Indeed, previous studies have shown that murine Irx5 co-immunoprecipitated with Gata3 and Trps1. When co-immunoprecipitation was done with these proteins with the Irx5 Asn166Lys mutation, there was markedly less binding to Trps1 and Gata3, demonstrating that Irx5 binding is reduced by Hamamy mutations and that Trps1 binding to Irx3 and Irx5 is likely affected in *Irx*3^*flox*/*flox*^/*Irx*5^−/−^/*Osx-Cre*^+^ mice (Bonnard et al., 2012).

*Gata*3^−/−^ mice are embryonically lethal from noradrenaline deficiency (Lim et al., 2000) and *Gata*3^−/−^ rescued embryos display cranial bone development defects, but *Gata*3^+/−^ mice appear to have no bone developmental abnormalities, which suggests that *Gata*3 is important for the development of skeletal tissues, but may not be involved in the regulation of bone mineralization gene expression (Lim et al., 2000, Pandolfi et al., 1995). Unfortunately, the expression of *Trps1* has not been reported in *Gata*3^+/−^ and *Gata*3^−/−^ mice (Lim et al., 2000). Future studies to understand how *Trps1* is regulated at specific stages of osteoblast mineralization, and if this defect is associated with the early lethality prior to puberty, will help us to determine the role of *Trps1* in the reduced mineralization of *Irx*3^*flox*/*flox*^/*Irx5*^−/−^/*Osx-Cre*^+^ mice.

Finally, why mice require loss of both IRX3 and IRX5 to develop reductions in mineralization similar to Hamamy patients who carry a nonsense mutation in the *IRX5* gene remains unclear (Gaborit et al., 2012). One notion to explain the similar reductions in bone mineralization between *Irx*3^*flox*/*flox*^/*Irx*5^−/−^/*Osx-Cre*^+^ mice and Hamamy patients is that deletion of both IRX3 and IRX5 in osteoblasts removes the ability to compensate for the loss of these proteins, whereas Hamamy *IRX*5 nonsense mutations influences IRX3 and IRX5 heterodimer formation and downstream mineralization function (Gaborit et al., 2012, He et al., 2009). More detailed analysis of Hamamy syndrome patients cells using a human induced pluripotent stem cell model may help demonstrate the dynamics of IRX3 and IRX5 interactions in Hamamy patient cells.

In conclusion, we identified a novel role for IRX3 and IRX5 in early cranial mineralization of osteoblastic cells. Furthermore, *Irx*3^*flox*/*flox*^/*Irx*5^−/−^/*Osx-Cre*^+^ mice displayed reduced bone mineralization without affecting early osteogenic gene expression. Finally, our finding that *Irx*3^*flox*/*flox*^/*Irx*5^−/−^/*Osx-Cre*^+^ mice have reduced osteoblastic mineralization indicates that IRX3 to IRX5 binding maintains an important role in Hamamy syndrome and understanding the role of IRX3 and IRX5 together will help provide insight into the roles of IRX proteins in other organs.

## 4 Methods

### 4.1 X-ray analysis of Jordanian and Turkish patients with Hamamy Syndrome

The Jordanian patients were originally described by Hanan Hamamy at Jordan University Hospital (Hamamy et al., 2007a). The Turkish family was diagnosed by Hülya Kayserili at the Medical Genetics Department of the Istanbul Medical Faculty (Bonnard et al., 2012, Hamamy et al., 2007a). Both sets of patients provided informed consent for radiographs to be published, and all studies have been approved by the local ethic commissions as described (Bonnard et al., 2012). Radiograph analysis was done on IRX5 Asn166Lys and IRX5 Ala150Pro patients and compared to control to assess osteopenia and craniofacial dysmorphisms. DEXA was used to measure area, BMD, and BMC of the lumbar spine and femoral neck, and to calculate *Z*-score. DEXA were performed just before puberty age (8–9 years old) and at adult age (19–20 years old).

### 4.2 Mice

All transgenic mouse studies were approved by and performed in accordance with the Institutional Animal Care and Use Committee at the University of California, San Francisco. *Irx*3^*flox*/*flox*^/*Irx*5^−/−^ mice were generated as described (Gaborit et al., 2012). To create tissue specific *Irx*3 knockout in osteoblast-lineage cells, we crossed *Irx*3^*flox*/*flox*^/*Irx*5^−/−^ mice with *Osx-Cre* hemizygous transgenic mice (Jackson Laboratory; strain: B6·Cg-Tg(Sp7(Osx)-tTA,tetO-EGFP/cre)1Amc/J Jackson ID: 6361) to generate *Irx*3^*flox*/*flox*^/*Irx*5^−/−^/*Osx-Cre*^+^ mice (Rodda & McMahon, 2006). *Osx-Cre*^+^ transgenic mice were found to have early lethality (Supplemental Fig. 1C) and a modest newborn bone phenotype that subsided by 3–4 weeks of age, which has been previously reported (Wang et al., 2015). We obtained 43% viability of *Irx*3^*flox/flox*^/*Irx*5^−/−^/*Osx-Cre*^+^ at birth. Since the *Irx*3 and *Irx*5 loci are close together, these alleles segregate independently only at low frequency (Supplemental Fig. 1) and so these genotypes were not analyzed. In addition, we found that *Osx-Cre*^+^ mice have a skeletal mineralization deficiency at birth, consistent with recent reports (Wang et al., 2015, Huang and Olsen, 2015). Thus, all experiments include these mice as a control in our analyses. For gene expression analysis, breeding pairs of *Irx*3^*flox*/+^/*Irx*5^+/−^/*Osx-Cre*^+^ crossed with *Irx*3^*flox*/+^/*Irx*5^+/−^/*Osx-Cre*^+^ mice were used with no detectable phenotypic differences observed in Osx-Cre single and double transgenic mice. All data are from both male and female mice.

### 4.3 Alizarin red and alcian blue staining of skeletons

Newborn mice and 3–4 week old mice of both sexes were euthanized and prepped for alizarin red and alcian blue skeletal staining (Ovchinnikov, 2009) by fixing in 100% ethanol for 24 h. Samples were then switched to acetone (Sigma-Aldrich) for an additional 24 h. Once fixed, samples were stained with final concentration of 5% glacial acetic acid, 0.5% alizarin red S (Sigma-Aldrich), 0.9% alcian blue 8GX (Sigma-Aldrich) in ethanol for 3 h at 37 °C and then at room temperature for 24 h. Samples were then placed in 1% KOH (Amresco) for 3 h and replaced with fresh KOH until non-bone tissue was transparent. Samples were then replaced with increasing concentrations of glycerol and photographed with a Leica MZFLIII dissection microscope with Diagnostic Instruments 14.2 Color Mosaic camera for newborn samples. 3–4 week old samples were photographed with a Nikon E5200 without a microscope.

### 4.4 Histology

Newborn skulls were skinned and fixed in neutral buffered formalin for at least 48 h and then replaced with 70% ethanol for at least 24 h. Skull tissues were paraffin embedded and sectioned. Skulls were then cut at the midline and then stained with hematoxylin & eosin, using standard protocols (J. David Gladstone Institutes Histology Core).

### 4.5 Bone densitometry and microCT imaging

DEXA was used to measure mouse whole-body BMD and BMC. Mice were anesthetized with inhaled isofluorane (1.5% to 2% in oxygen) and scanned on a GE Lunar Piximus2 (Piximus). Newborn mice that underwent whole-mouse microCT scans were sacrificed and stored in 70% ethanol before scanning. *Ex vivo* images were obtained on a Scanco vivaC*T*-40 microCT scanner (SCANCO) at an X-ray energy of 55 kV, with sigma 0.8/support 1/threshold 120 (103.7 mg HA/cm^3^), a voxel size of 76 μm, and integration times of 200 ms for whole-body images.

### 4.6 RNA isolation, cDNA synthesis, and qPCR

Whole calvaria or dissected calvarial tissues were placed in Trizol (Invitrogen) and homogenized using a Powergen 125 homogenizer (Fisher). RNA was isolated using chloroform extraction for whole calvaria or by Picopure RNA isolation columns for dissected calvaria (Life Technologies). Purified mRNA was then used as a template to synthesize cDNA with oligo dT primers with the Superscript III (Invitrogen) kit as described (Cain & Manilay, 2013). qPCR expression analysis was performed using TaqMan primers for qPCR reactions (Supplementary Table 1) on a Viia7 real-time thermocycler (Applied Biosystems) run in 5-μl sample volumes in triplicate or preamplified using Fludigm preamplication qPCR mix and assayed using Fluidigm dynamic array IFC qPCR plates (Fludigm). All expression values were normalized to *Gapdh* levels.

### 4.7 Statistics

Differences between the means of biological replicates for all analyses were calculated using two tailed Student's *T*-test (GraphPad Prism. La Jolla, *CA*). Analyses were considered statistically significant if *p* ≤ 0.05.

The following are the supplementary data related to this article.

**Supplementary Table 1:**
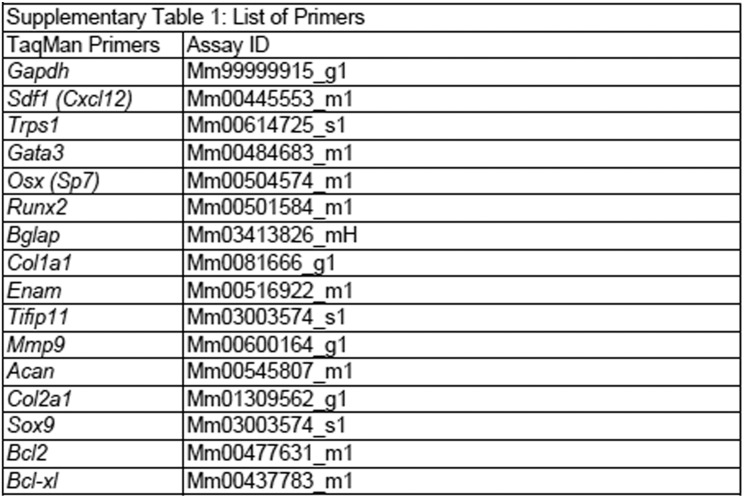
List of TaqMan primers used for gene expression studies.

## Conflict of interest statement

Edward Hsiao receives funding from Clementia Pharmaceuticals for an unrelated clinical trial.

## Figures and Tables

**Table 1:** Hamamy Patient *Z*-scores.

IRX5 Asn166Lys	Patient 1		Patient 2	
Age (years)	9	20	8	19
Z-score lumbar spine	− 1.5	− 1.4	− 3.7	− 2.7
Z-score femoral neck	− 2.2	Greater than − 1	N.D.	Greater than − 1
